# Value of deep learning models based on ultrasonic dynamic videos for distinguishing thyroid nodules

**DOI:** 10.3389/fonc.2022.1066508

**Published:** 2023-01-17

**Authors:** Chen Ni, Bojian Feng, Jincao Yao, Xueqin Zhou, Jiafei Shen, Di Ou, Chanjuan Peng, Dong Xu

**Affiliations:** ^1^ The Second Clinical School of Zhejiang Chinese Medical University, Hangzhou, China; ^2^ Key Laboratory of Head and Neck Cancer Translational Research of Zhejiang Province, Hangzhou, China; ^3^ Department of Ultrasonography, The Cancer Hospital of the University of Chinese Academy of Sciences (Zhejiang Cancer Hospital), Institute of Basic Medicine and Cancer, Chinese Academy of Sciences, Hangzhou, Zhejiang, China; ^4^ Clinical Research Department, Esaote (Shenzhen) Medical Equipment Co., Ltd., Xinyilingyu Research Center, Shenzhen, China; ^5^ Cancer Hospital of the University of Chinese Academy of Sciences (Zhejiang Cancer Hospital), Hangzhou, China; Key Laboratory of Head and Neck Cancer Translational Research of Zhejiang Province, Hangzhou, China

**Keywords:** ultrasound, thyroid nodules, deep learning, distinguishing, video

## Abstract

**Objective:**

This study was designed to distinguish benign and malignant thyroid nodules by using deep learning(DL) models based on ultrasound dynamic videos.

**Methods:**

Ultrasound dynamic videos of 1018 thyroid nodules were retrospectively collected from 657 patients in Zhejiang Cancer Hospital from January 2020 to December 2020 for the tests with 5 DL models.

**Results:**

In the internal test set, the area under the receiver operating characteristic curve (AUROC) was 0.929(95% CI: 0.888,0.970) for the best-performing model LSTM Two radiologists interpreted the dynamic video with AUROC values of 0.760 (95% CI: 0.653, 0.867) and 0.815 (95% CI: 0.778, 0.853). In the external test set, the best-performing DL model had AUROC values of 0.896(95% CI: 0.847,0.945), and two ultrasound radiologist had AUROC values of 0.754 (95% CI: 0.649,0.850) and 0.833 (95% CI: 0.797,0.869).

**Conclusion:**

This study demonstrates that the DL model based on ultrasound dynamic videos performs better than the ultrasound radiologists in distinguishing thyroid nodules.

## Introduction

Thyroid nodules are common diseases in humans, with an incidence as high as 68% (1). These nodules are mostly benign with a good prognosis, requiring only long-term follow-up and monitoring in the absence of intervention. However, approximately 7–15% of thyroid nodules are diagnosed as malignant, and some malignant nodules may cause local invasion, vocal cord paralysis, distant metastasis, postoperative recurrence, etc. (2) Therefore, the early detection of thyroid nodules and distinguishing benign from malignant thyroid nodules contributes to a reasonable treatment regime for patients and effectively reduces their prognostic risks.

Non-invasive, simple, convenient, and repeatable ultrasonography is currently the most commonly used imaging method for screening thyroid nodules in clinical practice (3), and with the upgrading of this method, the detection rate of thyroid nodules has also increased (4) The 2017 Thyroid Imaging Reporting and Data System (ACR TI-RADS) (5) is a kind of ultrasound reporting system that enables a standardized assessment for the grade of malignancy of thyroid nodules, which effectively standardizes the ultrasound report description and optimizes the treatment and management measures of thyroid nodules, thus improving the accuracy in distinguishing benign from malignant thyroid nodules. However, the clinical diagnosis of thyroid nodules based on ACR TI-RADS classification is time consuming and often affected by the subjective experience and interpretation of the examining physician, providing unstable accuracy (6); hence, such a diagnosis should be further confirmed by other examinations. The American Thyroid Association (7) recommends ultrasound-guided fine-needle aspiration cytology (US-FNA) as a qualitative diagnosis of suspicious thyroid nodules. This approach is simple and repeatable with fewer complications and is considered to be the gold standard for the diagnosis of thyroid nodules. However, it has been pointed out (1, 8) that US-FNA has a misdiagnosis rate of 15–30% and does not fully clarify the pathological nature of the examined nodules. To this end, there is an urgent need for a more accurate and safe method to distinguish benign from malignant thyroid nodules.

In recent years, artificial intelligence (AI) has been more widely used in medicine with the rapid development of computer technology, making many impossible tasks in medicine in the past viable (9–13). As the core technique of AI research, deep learning is primarily used for classification and prediction, which achieves the purpose of diagnosis through classifiers by extracting ultrasound image features, texture analysis, and image segmentation (14, 15).This approach can reveal various disease characteristics that are not identified by humans in daily practice (16, 17). Some studies (18–20) have demonstrated that deep learning(DL) models can help ultrasound radiologists effectively judge suspicious thyroid nodules at present and distinguish them more accurately. Li (8) used the DL model to discriminate benign from malignant thyroid nodules. Sui (1) developed the Thynet model, which helps ultrasound radiologists distinguish benign and malignant thyroid nodules more effectively in the absence of fine-needle aspiration. However, in existing studies, DL models are all based on ultrasonic static images (21), which, although they have high accuracy, cannot completely simulate the clinical settings. In real clinical settings, ultrasound radiologists often need to find nodules and judge their grade of malignancy during dynamic scanning of the thyroid gland, but a static image often does not contain all information of the nodules. Therefore, this study will focus on whether a deep learning model more in line with clinical settings can be established based on dynamic videos to distinguish benign from malignant thyroid nodules.

Based on the above discussion, five deep learning(DL) models were adopted in this study to distinguish benign and malignant thyroid nodules based on ultrasound dynamic videos, and their clinical application value was explored.

## Methods

### Data source

With informed consent exempted in this study, patient data were analyzed anonymously, and all personal information was removed from the final results. The study was approved by the ethics committee of the hospital and conducted in accordance with the Declaration of Helsinki. In this study, we retrospectively collected the thyroid nodule cases receiving surgery from January 2020 to December 2020 from the ultrasound database of Zhejiang Cancer Hospital. All patients underwent routine ultrasonography in the Ultrasound Department of the hospital, with complete dynamic ultrasound video data available. The ultrasonography was performed using Philips iU 22 and GE E 9 color Doppler ultrasound diagnostic instruments equipped with 9L-4 linear array probe with a frequency of 10–12 MHz. All patients were examined in the supine position with the neck straight. Both sides of the neck were fully exposed, and the thyroid gland was scanned in the transverse and longitudinal axes. The acquisition of ultrasound dynamic videos was completed by two ultrasound radiologists with more than 5 years’ experience.

### Inclusion and exclusion criteria

The criteria for nodule cases included in this study were as follows: ① patients aged > 18 years; ② underwent total or unilateral thyroidectomy in our hospital; ③ clear pathological results within 1 month after surgery; ④ clear ultrasound dynamic video images retained. The exclusion criteria were as follows: ① nodule diameter < 3 mm; ② pathological results were not available or were unclear; ③ video images did not show nodules clearly or showed them in poor quality; ④ ultrasonography findings showed inconsistent site or size of the lesion; ⑤ received chemotherapy and/or radiotherapy such as iodine 131 before ultrasonography. Criteria for dynamic video saving: ① the entire process from the appearance to the disappearance of the nodule was completely acquired; ② the shape and surroundings of the nodule were fully exposed during the acquisition; ③ the length of videos acquired was not less than 10 s. All video data were stored in DICOM format on the hard disk of the machine.

All cases enrolled had clear postoperative pathology. Postoperative pathological data were provided by the Department of Pathology, Zhejiang Cancer Hospital. All pathological assessments were performed based on HE-stained whole-slide images by using The Bethesda System For Reporting Thyroid Cytopathology (TBSRTC) (9). Only the nodules with clear pathological types of Bethesda Class I, II, or VI were included for training.

### Data classification and processing

**Figure 1:**
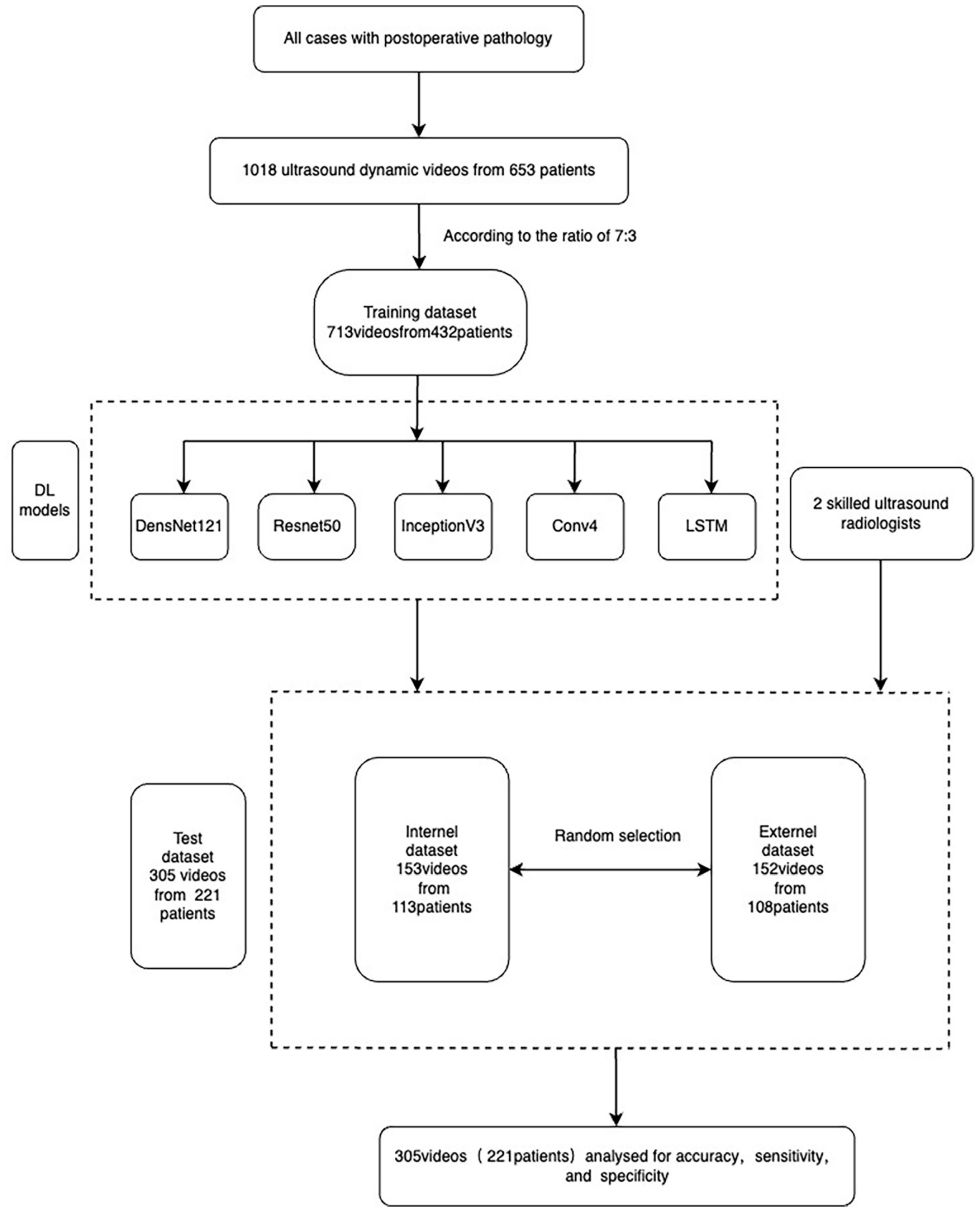
Flow chart for inclusion and exclusion criteria.

In this study, thyroid ultrasound dynamic videos of 657 patients were collected. With some unsatisfactory excluded against the exclusion criteria, we obtained ultrasound dynamic videos of a total of 1018 nodules. According to the pathological results, we divided these videos into benign or malignant nodule groups. In this study, the total videos were randomly divided into either training or test sets in a ratio of 7:3, and the test set was subdivided into internal or external test sets in a ratio of 1:1. A total of 713 videos were used for model training—153 videos for internal and 152 videos for external tests (**Figure 1**). Two attending ultrasound radiologists with more than 5 years’ working experience read the included nodule videos separately. In case of any doubt regarding the results, the chief (associate chief) ultrasound radiologist was available for assistance, and the comments from this ultrasound radiologist should prevail.

**Figure 2:**
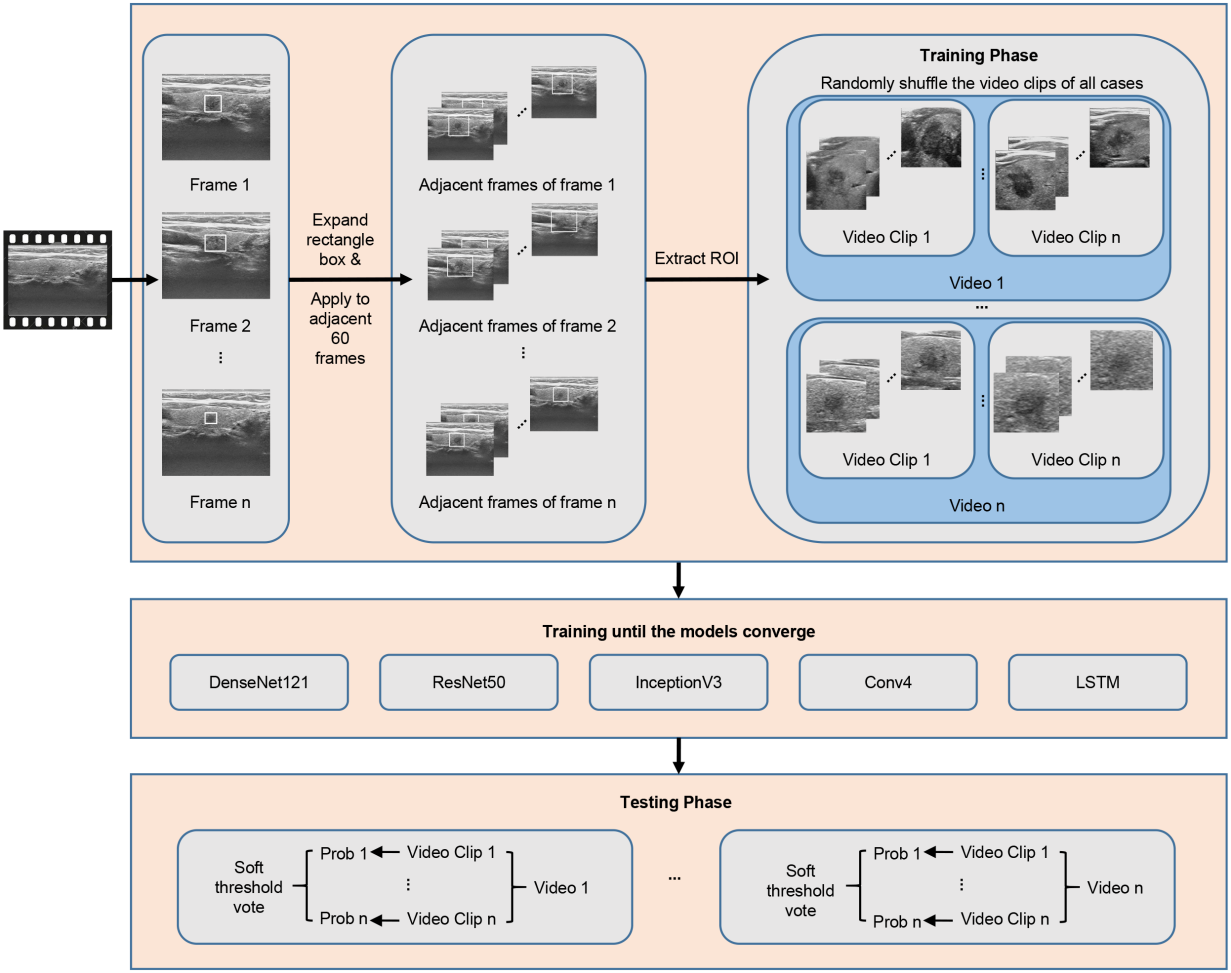
Overall flow chart for DL models deployment.

For the processing of thyroid ultrasound videos, we utilized Python language-based Pydicom and OpenCV packages to read the raw data in Digital Imaging and Communications in Medicine (DICOM). After original video data were available, the nodule region was extracted first. The extraction of the nodule region in the video is difficult because there are varying thyroid nodule images from 300 to 3600 frames in the videos in this data set calculated based on the varying length of the video from 10 to 120 seconds and a frame rate of 30 frames per second (**Figure 2**). It would be both time and labor consuming for clinicians to mark each frame of the video. Therefore, the clinician herein selected 1–10 frames with clear nodule features for each video to mark the nodule region in a rectangle box. Using this approach has two advantages: first, the clinician does not need to spend a lot of time marking the nodule region in the video because only 1–10 frames need to be marked; second, after the nodule region is marked on a specific frame, the sequence containing a total of adjacent 60 frames of the marked frame is selected and stitched on the channel dimension as a video clip.

**Figure 3:**
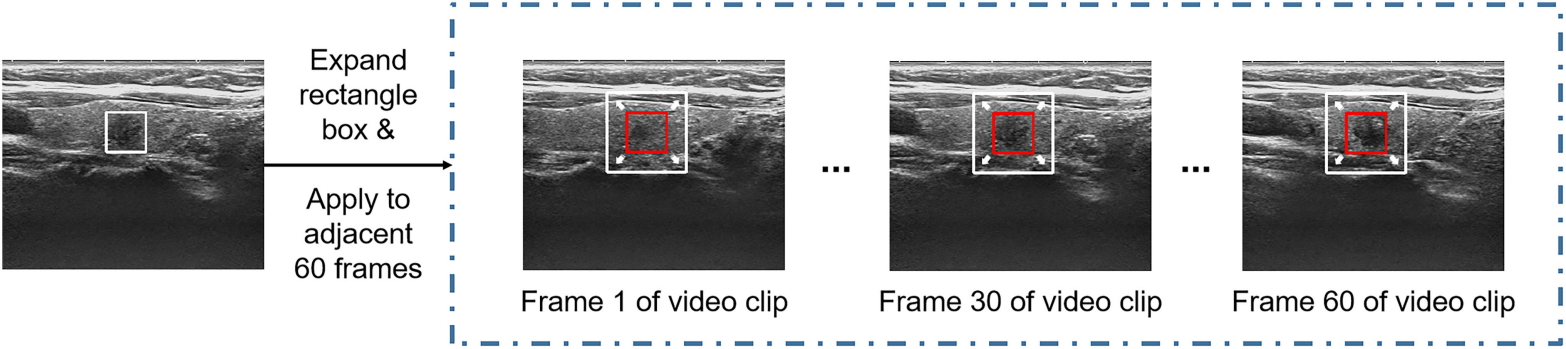
Nodule position on one of the frames was only marked for each video clip, and the marking box for the marking on all frames in the video clip was appropriately expanded.

Because the nodule position changed little within 2 seconds, the nodule region marking on a specific frame was applicable to its adjacent frames, thus substantially reducing the physician’s marking time. One to ten video clips can be obtained by marking one video. To cover more important tissues and make the model more robust, the rectangle box of the marked nodule region was expanded herein by 1.5 times along the four directions (i.e., up, down, left, right) so that the marked region can cover more nodules and important features (22, 23) (**Figure 3**). Data augmentation, including random translation (if the bounding box exceeded the frame boundary, it will be discarded), random cropping, and adding Gaussian noise with a mean value of 0 and a variance of 1, was used to increase the diversity of data and avoid the risk of overfitting.

For the selection of static images in the comparative experiment, the frames with marked nodule regions were trained and tested as static images herein. All data were standardized by subtracting the overall mean and dividing by the variance, and they were structured into TFRecord format to improve the utilization of graphics processing units and accelerate training.

We utilized five DL models—DenseNet121 (24), ResNet50 (25), InceptionV3 (26), long short-term memory (LSTM) (27) and a self-built small network named Conv4—to model the extracted videos by category by analyzing and comparing the parameters and structures of these networks. DL can construct the correct mapping from nodule video data to classification of benign and malignant nodules based on a large amount of data by simulating the feature-extraction method of clinicians. The five networks used herein have their own advantages. DenseNet121 contains dense connections and has the ability to efficiently extract features based on fewer parameters; InceptionV3 can extract more complex features due to its network width; ResNet50 can prevent exploding or vanishing gradients *via* its unique skip-connection; LSTM is a recurrent neural network that excels at classifying, processing, and predicting long sequence data.; to prevent network overfitting due to a too-small dataset, a small network containing only four convolutional layers was constructed herein, named Conv4. All video clips were resized to 224 × 224 × 60 to meet the network’s requirement for the same input size before they are input into the network. The five networks were first iteratively trained and back-propagated to adjust the parameters based on the training set, and the parameters were fixed after the network converged and tested on the internal and external test sets. In the network training, the video clips from various videos were mixed and randomly input into the networks; in contrast, in the test stage, to avoid the video clips from the same nodule video present in both training and test sets, we utilized the networks to test the video clips from the same video separately and determined the final classification of this video through soft voting.

To compare the effects of different networks and training mechanisms on the identification more effectively, the hyper-parameters of all experimental groups were unified herein using an optimized algorithm Adam, with the momentum value set to 0.9, the initial learning rate set to 0.001, 100 epochs run in each experiment, the Batchsize set to 128, and the Dropout value set to 0.5. All experiments herein were implemented by the Keras 2.1.5 and Tensorflow 1.6.0 frameworks under the Ubuntu16.04 system, using a host equipped with Intel (R) Core (TM) i7-8700@3.2G and NVIDIA TITAN V 12GB GPU.

### Statistical analysis

The area under the receiver operating characteristic curve (AUROC) was used herein to demonstrate the ability of each DL model to distinguish benign from malignant thyroid nodules based on videos. In addition, the comprehensive performance of networks was evaluated based on the sensitivity, specificity, positive predictive value (PPV), negative predictive value (NPV), and accuracy, and the 95% CI of each index was calculated using the DeLong method. Calculation of all indexes and plotting of ROC curves were both performed with Python language-based matplotlib and sklearn packages.

Moreover, the kappa coefficient was calculated using the criteria of Landis (28) to evaluate the agreement between the result predicted by the network and the pathological gold standard. All calculated *P* values for 95% CIs were less than 0.05 and were statistically significant.

## Results

In this study, thyroid ultrasound dynamic videos of 653 patients were collected. The training test set included 90 males and 347 females, with a mean age of 46.59 ± 11.56 years and a mean lump size of 12.85 ± 11.54 mm. The internal test set included 25 males and 88 females, with a mean age of 47.80 ± 12.31 years and a mean lump size of 14.25 ± 10.56 mm. The external test set included 29 males and 79 females, with a mean age of 47.30 ± 11.89 years and a mean lump size of 14.66 ± 11.13 mm (**Table 1**).

**Table 1:** Baseline characteristics.

	Training dataset n=437	Internal dataset n=113	External dataset n=108
Age (mean ± SD)	46.59 ± 11.56(23,83)	47.80 ± 12.31 (23, 74)	47.30 ± 11.89 (18, 75)
18–30	46(11%)	12 (11%)	10 (9%)
31–50	216(49%)	51 (45%)	51 (47%)
> 50	175(40%)	50 (44%)	47 (44%)
Sex			
Male	90(21%)	25 (22%)	29 (27%)
Female	347(79%)	88 (78%)	79 (73%)
Lump size (mm)	12.85 ± 11.54(3,83)	14.25 ± 10.56 (4,47)	14.66 ± 11.13 (4,58)
3–10	269(62%)	64 (57%)	57 (53%)
11–20	92(21%)	24 (21%)	28 (26%)
> 20	76(17%)	25 (22%)	23 (21%)

**Tables 2**–**4** and **Figure 4** show the comparative performance of the five CNN models (Conv4, DenseNet121, ResNet50, InceptionV3 and LSTM) *vs*. the ultrasound radiologists in distinguishing benign from malignant thyroid nodules. In the internal test set, the area under the receiver operating characteristic curve (AUROC) was 0.929(95% CI: 0.888,0.970)for the best-performing model LSTM, 0.927(95% CI: 0.885,0.969)for Conv4,0.876(95% CI: 0.823,0.928)for DenseNet121, 0.896(95% CI: 0.848,0.945) for ResNet50, and0.917(95% CI: 0.873,0.961)for InceptionV3. Two radiologists interpreted the small video with AUROC values of 0.760 (95% CI: 0.653,0.867) and 0.815 (95% CI: 0.778,0.853), respectively. The five DLs all performed better than the ultrasound radiologists in identifying thyroid nodules. In terms of accuracy, sensitivity, and specificity, the best-performing DL had an accuracy of 91.3%(95% CI: 0.868,0.958), and both radiologists had an accuracy of 79.3% (95% CI: 0.717,0.863). The sensitivity of the best-performing DL model was94.5%(95% CI: 0.902,0.988), and the sensitivity of the radiologists ‘ readings was 82.4% (95% CI: 0.737, 0.911) and 85.1% (95% CI: 0.770,0.932), respectively. For specificity, the best-performing DL model was 85.4%(95% CI: 0.745,0.962, and the specificity of the radiologists was 73.0% (95% CI: 0.587,0.873) and 67.5% (95% CI: 0.524,0.826), respectively. It can be seen that in terms of accuracy, sensitivity, and specificity, the DL model performed better than the radiologists. All five models were highly stable, with a kappa value exceeding 0.05 and a *P* value less than 0.5. In the external test set, the best-performing DL model had AUROC values of 0.896(95% CI: 0.847,0.945), and two ultrasound radiologist had AUROC values of 0.754 (95% CI: 0.649,0.850) and 0.833 (95% CI: 0.797,0.869). In terms of accuracy, sensitivity, and specificity, the best-performing DL algorithm had an accuracy of 91.9%(95% CI: 0.875,0.963), and the two ultrasound radiologist had an accuracy of 80.4% (95% CI: 0.728,0.879) and 82.2% (95% CI: 0.750,0.895). The sensitivity of the best-performing DL algorithm was 97.4%(95% CI: 0.945,1.003), and the sensitivity of the ultrasound radiologists ‘ readings was 82.7% (95% CI: 0.745, 0.910) and 80.2% (95% CI: 0.716,0.889), respectively. For specificity, the best-performing DL algorithm was 87.9% (95% CI: 0.767, 0.990), and the specificity of the ultrasound radiologists was 73.1% (95% CI: 0.560,0.910) and 88.7% (95% CI: 0.762,1.007), respectively, demonstrating that the DL models trained based on dynamic videos performed better than the ultrasound radiologists. In addition, we compared an image screenshot that showed the nodule position and morphology most clearly in each video with the dynamic video. In the external test set, the AUROC of the static image was 0.815 (95% CI: 0.778, 0.853), the accuracy was 74.8% (95% CI: 0.705, 0.790), the sensitivity was 74.0% (95% CI: 0.694, 0.787), and the specificity was 78.2% (95% CI: 0.689, 0.880). In the internal test set, the AUROC of the static image was 0.833 (95% CI: 0.797, 0.869), the accuracy was 77.0% (95% CI: 0.729, 0.810), the sensitivity was 77.6% (95% CI: 0.731, 0.820), and the specificity was 73.9% (95% CI: 0.636, 0.843). It can be concluded that the diagnostic performance of the static image is not as good as that of the dynamic video in both the internal and external test sets. This finding indicates that the DL model trained based on the dynamic video has prominent advantages over the static image in distinguishing benign and malignant thyroid nodules in terms of accuracy, sensitivity, and specificity.

**Figure 4:**
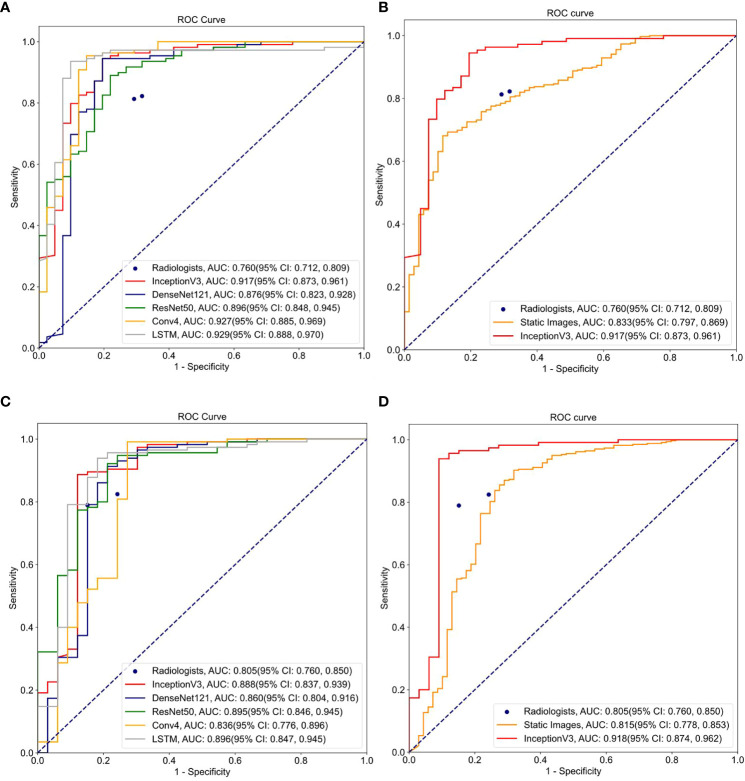
ROC curves for different models and ultrasound radiologists’ interpretations: **(A)** ROC curve for 5 DL learning models trained on video clips using internal test set; **(B)** ROC curve for models trained on video clips and static images using internal test set; **(C)** ROC curve for 5 DL models trained on video clips using external test set; **(D)** ROC curve for models trained on video clips and static images using external test set.

**Table 2:** Diagnostic performance of DL model in internal test set.

	Conv4	ResNet50	InceptionV3	DenseNet121	LSTM	Picture
Accuracy (95% CI)	0.913 (0.868,0.958)	0.853 (0.797,0.910)	0.873 (0.820,0.927)	0.880 (0.828,0.932)	0.913 (0.868,0.958)	0.770 (0.729, 0.810)
Sensitivity (95% CI)	0.936 (0.890,0.982)	0.881 (0.820,0.942)	0.899 (0.843,0.956)	0.908 (0.854,0.962)	0.945 (0.902,0.988)	0.776 (0.731, 0.820)
Specificity (95% CI)	0.854 (0.745,0.962	0.780 (0.654,0.907)	0.805 (0.684,0.926)	0.805 (0.684,0.926)	0.829 (0.714,0.944)	0.739 (0.636, 0.843)
PPV (95% CI)	0.944 (0.901,0.988)	0.914 (0.861,0.968)	0.925 (0.874,0.975)	0.925 (0.875,0.975)	0.936 (0.891,0.982)	0.936 (0.907, 0.965)
NPV (95% CI)	0.833 (0.721,0.946)	0.711 (0.579,0.844)	0.750 (0.622,0.878)	0.767 (0.641,0.894)	0.850 (0.739,0.961)	0.402 (0.316, 0.487)
AUROC (95% CI)	0.927 (0.885,0.969)	0.896 (0.848,0.945)	0.917 (0.873,0.961)	0.876 (0.823,0.928)	0.929 (0.888,0.970)	0.833 (0.797, 0.869)
K (Kappa)	0.783	0.642	0.688	0.702	0.780	0.386
F1	0.940	0.897	0.912	0.917	0.941	0.848
P-value	All <0.05					

**Table 3:** Diagnostic performance of DL model in external test set.

	Conv4	ResNet50	InceptionV3	DenseNet121	LSTM	Picture
Accuracy (95% CI)	0.919 (0.875,0.963)	0.905 (0.858,0.953)	0.905 (0.858,0.953)	0.863 (0.807,0.919)	0.912 (0.867,0.958)	0.748 (0.705, 0.790)
Sensitivity (95% CI)	0.974 (0.945,1.003)	0.930 (0.884,0.977)	0.913 (0.862,0.965)	0.870 (0.808,0.931)	0.939 (0.895,0.983)	0.740 (0.694, 0.787)
Specificity (95% CI)	0.727 (0.575,0.879)	0.818 (0.687,0.950)	0.879 (0.767,0.990)	0.839 (0.709,0.968)	0.818 (0.687,0.950)	0.782 (0.685, 0.880)
PPV (95% CI)	0.926 (0.879,0.972)	0.947 (0.906,0.988)	0.963 (0.928,0.999)	0.952 (0.912,0.993)	0.947 (0.906,0.988)	0.943 (0.916, 0.971)
NPV (95% CI)	0.889 (0.770,1.007)	0.771 (0.632,0.911)	0.744 (0.607,0.881)	0.634 (0.487,0.782)	0.794 (0.658,0.930)	0.380 (0.300, 0.460)
AUROC (95% CI)	0.836 (0.776,0.896)	0.895 (0.846,0.945)	0.888 (0.837,0.939)	0.860 (0.804,0.916)	0.896 (0.847,0.945)	0.815 (0.778,0.853)
K (Kappa)	0.750	0.733	0.744	0.634	0.749	0.368
F1	0.949	0.939	0.938	0.909	0.943	0.830
P-value	Al l<0.05					

**Table 4:** Performance of radiologists in reading videos.

	Internal Test Set		External Test Set	
	Radiologist 1	Radiologist 2	Radiologist 1	Radiologist 2
Accuracy (95% CI)	0.793 (0.717,0.868)	0.793 (0.717,0.868)	0.804 (0.728,0.879)	0.822 (0.750,0.895)
Sensitivity (95% CI)	0.824 (0.737,0.911)	0.851 (0.770,0.932)	0.827 (0.745,0.910)	0.802 (0.716,0.889)
Specificity (95% CI)	0.730 (0.587,0.873)	0.675 (0.524,826)	0.731 (0.560,0.901)	0.886 (0.762,1.007)
PPV(95% CI)	0.859 (0.778,0.940)	0.840 (0.757,0.923)	0.905 (0.839,0.972)	0.956 (0.907,1.004)
NPV (95% CI)	0.675 (0.530,0.820)	0.694 (0.543,0.845)	0.576 (0.407,0.744)	0.590 (0.435,0.744)
AUROC (95% CI)	0.760 (0.653,0.867)	0.815 (0.778,0.853)	0.754 (0.649,0.858)	0.833 (0.797,0.869)
K(Kappa)	0.543	0.531	0.511	0.587
F1	0.841	0.846	0.865	0.872
P-value	All < 0.05			

In addition, we performed a DeLong test to check the significance of the differences between the video-based DL model, the static image-based DL, and the video-based interpretation by the ultrasound radiologists using the external test set. As shown in **Table 4**, there was a significant difference (*p* < 0.0001) between the five video-based DL models and the static image-based model, as well as between the five video-based DL models and the ultrasound radiologists, further demonstrating that the video-based DL model has specific advantages over the ultrasound radiologists and the static image-based model in distinguishing benign from malignant thyroid nodules. For clinical use, we calculated the model weight, resident memory occupied by the model while running, and the inference speed of the CPU and GPU for each model. We calculated the total time required for each model to perform inference on all test and validation sets and took the average value. The results are shown in **Tables 5** and **6**.

**Table 5:** The *p*-values of the DeLong test for different methods in the external test set.

Methods	DenseNet121	ResNet50	InceptionV3	Conv4	LSTM	Static	Radiologists
**DenseNet121**	1.0000	0.1238	0.4995	0.2503	0.2034	<0.0001	<0.0001
**ResNet50**	0.1238	1.0000	0.3831	0.2099	0.3532	<0.0001	<0.0001
**InceptionV3**	0.4995	0.3831	1.0000	0.5802	0.2964	<0.0001	<0.0001
**Conv4**	0.2503	0.2099	0.5802	1.0000	0.4925	<0.0001	<0.0001
**LSTM**	0.2034	0.3532	0.2964	0.4925	1.0000	<0.0001	<0.0001
**Static**	<0.0001	<0.0001	<0.0001	<0.0001	<0.0001	1.0000	0.3559
**Radiologists**	<0.0001	<0.0001	<0.0001	<0.0001	<0.0001	0.3559	1.0000

**Table 6:** Model weight, model memory and inference time.

Methods	DenseNet121	ResNet50	InceptionV3	Conv4	LSTM
**Model Weight (GB)**	0.0296	0.0954	0.0878	0.0113	0.0248
**Resident Memory Usage (GB)**	1.1800	1.1622	1.1237	1.1388	3.7143
**Inference Time (s)/Clip on CPU**	0.1176	0.1075	0.0989	0.0769	0.2100
**Inference Time (s)/Clip on GPU**	0.0761	0.0521	0.0644	0.0431	0.0667

## Discussion

In this study, DL models were established based on ultrasound dynamic videos to distinguish between benign and malignant thyroid nodules more comprehensively. According to the results, the DL models used herein exhibited high accuracy, sensitivity, and specificity in distinguishing benign and malignant thyroid nodules in both the internal and external test sets, which outperformed the manual interpretation by the ultrasound radiologists.

In recent years, with higher health awareness among people and more advanced medical devices, the detection rate of thyroid nodules is increasing, making it one of the most prevalent diseases in humans (29). Ultrasonography is currently the preferred imaging modality for screening thyroid nodules in clinical practice, and the subsequent treatment plan is based on the ultrasonography result, either continued follow-up or surgery (30). Thyroid nodules are characterized by homogeneous echogenicity, indistinct borders, and varying morphology on different ultrasound instruments, so that ultrasound radiologists cannot accurately identify them and give results without differences (31). The manual interpretation herein was made independently by an ultrasound radiologist, following the advice of a senior physician if necessary. In the daily work environment, ultrasound radiologists are often required to independently interpret the found nodules. Therefore, the interpretation results are frequently different due to subjective factors and the work experience of the radiologists, and seeking advice from a senior physician represents a major expenditure of manpower and effort. DL models have advantages in addressing heterogeneity because they can extract engineering features of different nodules and are not limited by the benign and malignant nodule criteria adopted by physicians in identifying nodule features, thereby ensuring consistent results. Another advantage of DL models is that they can interpret the input nodules immediately, which saves a substantial amount of time and improves clinical work efficiency.

Previous studies have demonstrated the automated identification capability of DL models. Ko (32) designed three DL models to track and test 589 thyroid nodules, and the AUROC of the three DL models was 0.845, 0.835, and 0.850, respectively, which was not significantly different from the AUROC of 0.805–0.860 of the ultrasound radiologists, demonstrating that the DL models have a comparable diagnostic capability to ultrasound radiologists. In a multicenter study by Koh (12), 15375 thyroid nodule ultrasound images were trained with two DL models in order to compare the performance between the DL models and ultrasound radiologists in distinguishing benign and malignant thyroid nodules, and results showed that the DL models had similar sensitivity and higher specificity in identifying thyroid cancer patients compared with a group of skilled ultrasound radiologists. The DL models were also used herein for training based on dynamic videos and static images, respectively. From our findings, it can be seen that all DL models had a better capability in distinguishing benign and malignant nodules than the ultrasound radiologists, whether based on static images or dynamic videos, demonstrating the automated identification capability of DL models. Sui (1) simulated real clinical settings and distinguished benign and malignant thyroid nodules with 500 dynamic videos after the first round of static image reading. The AUROC increased from 0.862 to 0.873, and then increased to 0.877 after DL models were adopted to support the interpretation. In this study, dynamic videos were only included in the manual interpretation stage so as to improve the ultrasound radiologists’ accuracy by providing more information about nodules. The models were trained based on dynamic videos to identify thyroid nodules herein, and the results showed that DL models performed better than the ultrasound radiologists.

**Figure 5:**
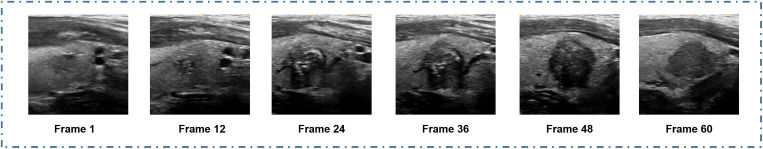
Ultrasound video showing more about the nature of the nodule than the static image: Frames 1 and 36 show the different morphology of the calcification in the nodule; frame 36 shows only a small part of punctate calcification in the periphery; frame 1 illustrates clear annular calcification in the periphery of the nodule; frame 48 shows aspect ratio imbalance of the nodule (i.e., greater height than length); frame 60 illustrates the solid composition of the nodule. This video completely shows three different malignancy features of the nodule: calcification, aspect ratio imbalance, and solid composition.

The models selected in the above studies (11, 13) were all trained based on static ultrasound images. However, in actual clinical practice, static images cannot completely simulate the clinical setting because they often do not cover the suspicious features of all nodules (33–35), potentially leading to some suspicious nodule features being missed, thus affecting the accuracy of the results. To display more complete feature information in static images as much as possible, ultrasound radiologists will spend more time and energy. Therefore, we plan to eliminate the above disadvantages by training DL models based on dynamic ultrasound videos (**Figure 5**). From our findings, it can be seen that models demonstrated better specificity, sensitivity, and accuracy in nodule identification in dynamic videos than in static images. DL models trained based on videos and images both performed better than the ultrasound radiologists in diagnosing nodules.

The thyroid ultrasound videos used in this study were produced by several different types of ultrasound instruments, contributing to increased data diversity. The thyroid nodules included herein were not limited to pathological type; this approach improved the clinical application value to some extent. There are also significant limitations in this study. First, the total sample size is small. Only the ultrasound video data within one year in one hospital are included, and most of the patients in the cohort are from the same or similar regions and cannot represent the entire population, causing errors in the study’s results. Therefore, multicenter studies with more data are required in the future. Second, the malignant cases in the data source center account for a high proportion, and the ratio of benign to malignant cases is not, therefore, as close as 1:1; this issue could lead to differences in the diagnostic performance of DL models for benign and malignant nodules. Therefore, more benign cases will be included in future studies. This study has potential clinical value. On the one hand, the DL models used herein can help ultrasound radiologists judge benign and malignant thyroid nodules in a close-to-real clinical setting, contributing to the development of a subsequent treatment plan and provision of accurate and timely medical services for patients. On the other hand, the accurate identification of benign and malignant thyroid nodules may avoid unnecessary fine-needle aspiration and surgery, reducing excessive medical treatment and waste of medical resources. Finally, medical resources are unbalanced between urban and rural areas in China and around the world, and the DL models adopted herein are helpful in solving this situation. In summary, the differential diagnosis of benign and malignant thyroid nodules by DL models based on ultrasound dynamic videos is worthy of further investigation and validation in prospective clinical trials.

## Data availability statement

The raw data supporting the conclusions of this article will be made available by the authors, without undue reservation.

## Ethics statement

The need for written informed consent was waived by th*e* Ethics Committee of Zhejiang Cancer Hospital due to the retrospective nature of the study. The study was conducted in accordance with the principles of the Declaration of Helsinki, and the study protocol was approved by the ethics committee of Zhejiang Cancer Hospital Because of the retrospective nature of the study, patient consent for inclusion was waived.

## Author contributions

DX contributed to the conception of the study. CN contributed to the data collection, organization and wrote the full article. BJF contributed to the construction of the deep learning model, revision of the manuscript, and the production of some figures in the manuscript. JCY contributed constructive suggestions on the construction of the depth model. XQZ contributed to the translation of the manuscript language. JFS, DO, CJP contributed to the collation of some of the data. All authors contributed to the article and approved the submitted version.
